# 2,5-Dihydroxyacetophenone Induces Apoptosis of Multiple Myeloma Cells by Regulating the MAPK Activation Pathway

**DOI:** 10.3390/molecules22071157

**Published:** 2017-07-11

**Authors:** Jeong-Hyeon Ko, Jae Hwi Lee, Sang Hoon Jung, Seok-Geun Lee, Arunachalam Chinnathambi, Sulaiman Ali Alharbi, Woong Mo Yang, Jae-Young Um, Gautam Sethi, Kwang Seok Ahn

**Affiliations:** 1 College of Korean Medicine, Kyung Hee University, 24 Kyungheedae-ro, Dongdaemun-gu, Seoul 02447, Korea; gokjh1647@gmail.com (J.-H.K.); apraxas0@hanmail.net (J.H.L.); seokgeun@khu.ac.kr (S.-G.L.); wmyang@khu.ac.kr (W.M.Y.); jyum@khu.ac.kr (J.-Y.U.); 2 Natural Products Research Institute, Korean Institute of Science and Technology, 679 Saimdang-ro, Gangneung, Gangwon-do 25451, Korea; shjung@kist.re.kr; 3 Department of Botany and Microbiology, College of Science, King Saud University, Riyadh 11451, Saudi Arabia; dr.arunmicro@gmail.com (A.C.); sharbi@ksu.edu.sa (S.A.A.); 4 School of Biomedical Sciences, Curtin Health Innovation Research Institute, Curtin University, Perth, WA 6009, Australia; 5 Department of Pharmacology, Yong Loo Lin School of Medicine, National University of Singapore, Singapore 117600, Singapore

**Keywords:** 2,5-dihydroxyacetophenone, multiple myeloma, MAPK, apoptosis

## Abstract

2,5-Dihydroxyacetophenone (DHAP) is an active compound obtained from *Radix rehmanniae preparata*, which is widely used as a herbal medicine in many Asian countries. DHAP has been found to possess anti-inflammatory, anti-anxiety, and neuroprotective qualities. For the present study, we evaluated the anti-cancer effects of DHAP on multiple myeloma cells. It was discovered that DHAP downregulated the expression of oncogenic gene products like Bcl-xl, Bcl-2, Mcl-1, Survivin, Cyclin D1, IAP-1, Cyclin E, COX-2, and MMP-9, and upregulated the expression of Bax and p21 proteins, consistent with the induction of G2/M phase cell cycle arrest and apoptosis in U266 cells. DHAP inhibited cell proliferation and induced apoptosis, as characterized by the cleavage of PARP and the activation of caspase-3, caspase-8, and caspase-9. Mitogen-activated protein kinase (MAPK) pathways have been linked to the modulation of the angiogenesis, proliferation, metastasis, and invasion of tumors. We therefore attempted to determine the effect of DHAP on MAPK signaling pathways, and discovered that DHAP treatment induced a sustained activation of JNK, ERK1/2, and p38 MAPKs. DHAP also potentiated the pro-apoptotic and anti-proliferative effects of bortezomib in U266 cells. Our results suggest that DHAP can be an effective therapeutic agent to target multiple myeloma.

## 1. Introduction

*Radix rehmanniae* is obtained from the root of the herbaceous plant *Rehmannia glutinosa* Libosch. It is a traditional Chinese medicinal herb and has been found to have biological properties like anti-inflammatory and wound-healing effects and the ability to attenuate diabetic nephropathy [1,2,3]. 2,5-Dihydroxyacetophenone (DHAP) is a one of the bioactive compounds isolated from *Radix rehmanniae preparata*, the steamed root of *Radix rehmanniae*, and has also been reported to have anti-inflammatory properties [4] through the modulation of nuclear factor kappa B (NF-κB) pathway-mediated inflammatory responses in the activated macrophages. Dysregulated inflammatory responses are important in a number of chronic diseases, including cancer [5,6,7,8], and we therefore postulate that DHAP may also exhibit promising anti-cancer properties.

The anti-tumor effect of cancer therapies is mainly brought about by the initiation of apoptosis in cancer cells [9,10,11,12,13,14]. Apoptosis is an evolutionarily conserved, intrinsic process of cell death that takes place in several physiological and pathological contexts [15]. The two main effector cascades involved in apoptosis are the intrinsic (mitochondrial) and extrinsic (death receptor) pathways [14,16,17,18]. The underlying mechanism(s) that cause an apoptosis response with cytotoxic therapy may be determined by the particular stimulus, and frequently have not been identified precisely. It has been suggested that several stress-inducible molecules, e.g., extracellular signal-regulated protein kinase (ERK), c-Jun N-terminal kinase (JNK), and NF-κB, may play a critical role in transmitting the apoptotic signals [19,20]. Mitogen-activated protein kinases (MAPKs) are serine-threonine protein kinases that are vital to the regulation of certain cellular properties, e.g., differentiation, cell growth and proliferation, and apoptosis. MAPKs consist of stress-activated JNK and p38, and growth factor-regulated ERK1/2 [11,21].

For this study, we attempted to determine if DHAP’s anti-cancer properties stem from the triggering of apoptosis in the multiple myeloma cell lines. We discovered that DHAP effectively inhibited multiple myeloma cell growth, induced apoptosis, and upregulated MAPK signaling pathways in vitro. In addition, DHAP exhibited synergistic effects when combined with the proteasome inhibitor, bortezomib. Our results therefore demonstrate the potential effectiveness of DHAP for the treatment of multiple myeloma.

## 2. Results

**Figure 1:**
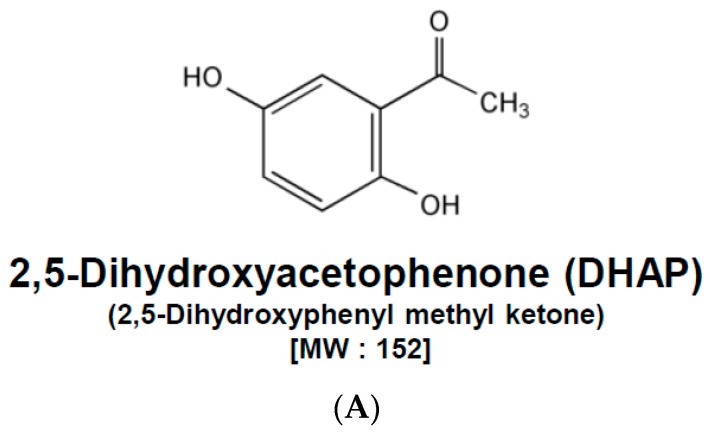

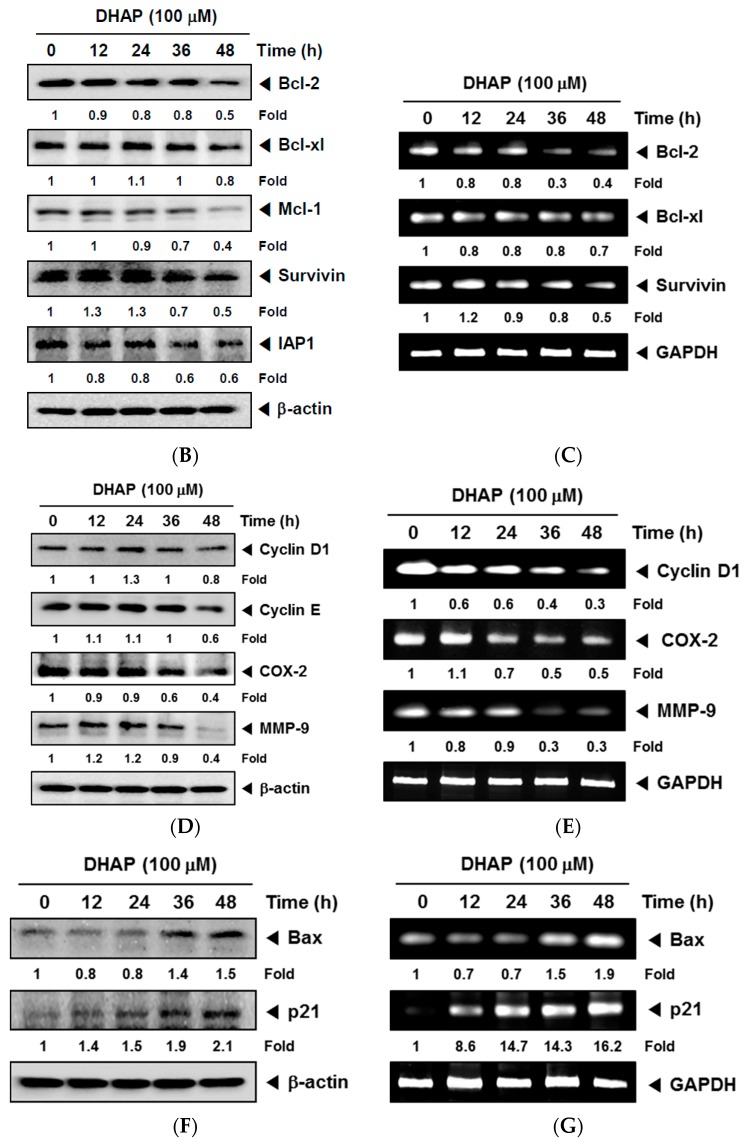
Effect of DHAP on anti-apoptotic and pro-apoptotic gene expression in U266 cells. (**A**) The chemical structure of 2,5-Dihydroxyacetophenone (DHAP). U266 cells were treated with 100 μM of DHAP for the time periods stated; (**B**) Bcl-xl, Bcl-2, Survivin, Mcl-1, and IAP1 protein levels were determined via Western blot analysis; (**C**) The gene expression levels of *Bcl-xl*, *Bcl-2*, and *Survivin* were determined by RT-PCR; (**D**) Cyclin D1, Cyclin E, COX-2, and MMP-9 protein levels were determined via Western blot analysis; (**E**) *Cyclin D1*, *COX-2* and *MMP-9* gene expression levels were determined by RT-PCR; (**F**) The protein levels of Bax and p21 were determined via Western blot analysis; (**G**) The gene expression levels of *Bax* and *p21* were determined by RT-PCR. The immunoblot was stripped and reprobed for β-actin to ensure equal protein loading. *GAPDH* mRNA expression was used as an internal control for normalization purposes. Densitometric quantitation in fold change of each band has been indicated below the gel. The results shown are representative of the three independent experiments.

For this study, we investigated the anti-tumor activity of DHAP in multiple myeloma cell U266 cells. The basic structure of DHAP is displayed in Figure 1A.

### 2.1. DHAP Modulates the Expression of Certain Proteins Connected to Apoptosis, Metastasis, and Proliferation

The cell survival proteins Bcl-2, Bcl-xl, Mcl-1, Survivin, and IAP1 have been linked to resistance to apoptosis [22,23], so we investigated the effect of DHAP on the constitutive expression of these proteins in U266 cells. It was noted that DHAP inhibited the expression of anti-apoptotic gene products in a time-dependent fashion (Figure 1B,C). In addition, DHAP down-regulated the expression of cell cycle proteins (Cyclin D1 and Cyclin E) and proteins relevant to metastasis (COX-2 and MMP-9) (Figure 1D,E). DHAP also induced the expression of pro-apoptotic proteins Bax and p21 in a time-dependent manner (Figure 1F,G). These results indicate that DHAP can trigger apoptosis by down-regulating proliferative, anti-apoptotic, and metastatic proteins, and by upregulating pro-apoptotic proteins in tumor cells.

### 2.2. DHAP Inhibits Cell Proliferation and Induces Apoptosis in U266 Cells

**Figure 2:**
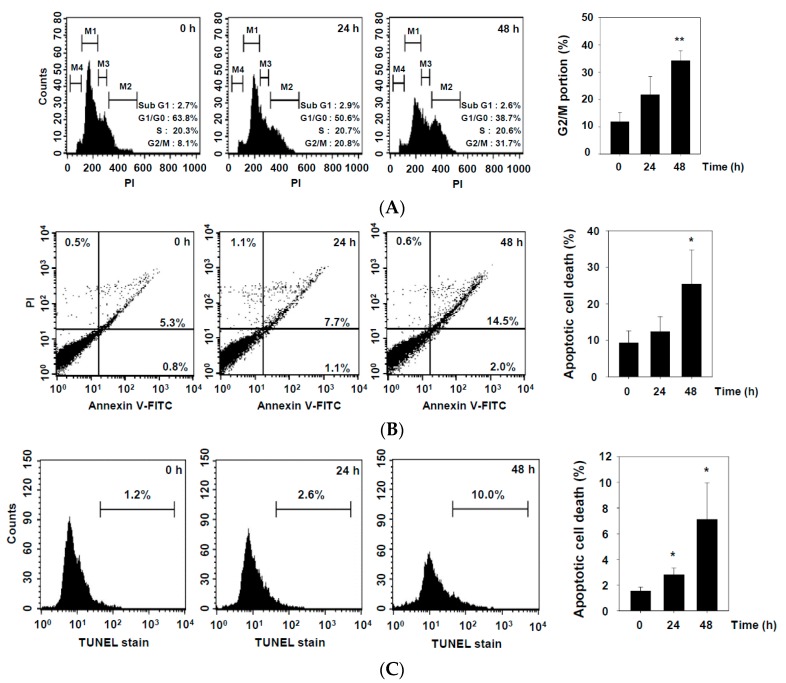

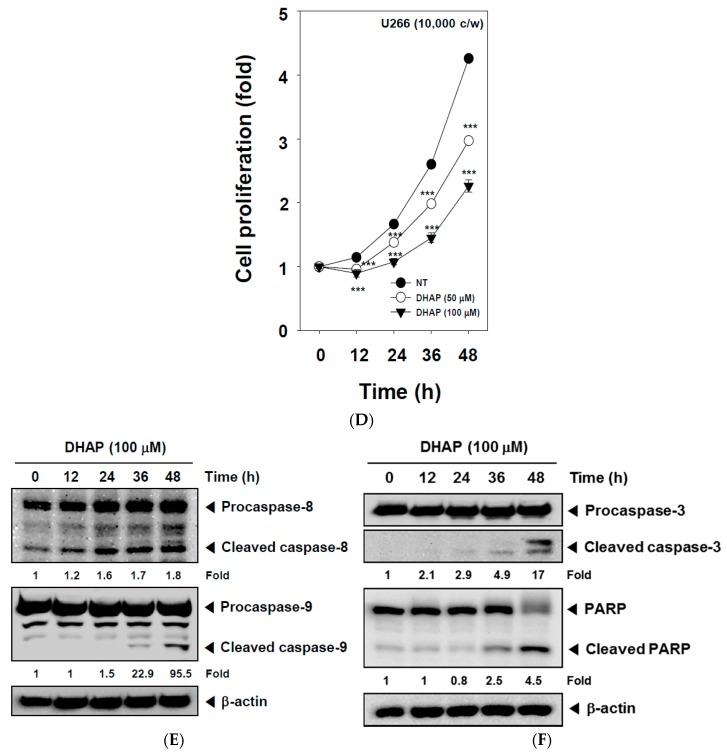
Effect of DHAP on apoptosis and proliferation of U266 cells. The cells were treated with 100 μM of DHAP for 24 h and 48 h. (**A**) Cellular DNA staining incorporating PI and flow cytometric analysis was performed to ascertain the cell cycle distribution; (**B**) The cells were incubated with an FITC-conjugated Annexin V, then examined for an early apoptotic effect with flow cytometry; (**C**) The cells were fixed and incubated with a TUNEL reaction solution, then examined for DNA fragmentation with flow cytometry; (**D**) U266 cells were treated with 50 and 100 μM of DHAP, then subjected to an MTT assay after 12, 24, 36, and 48 h, to enable cell proliferation to be examined; (**E**) U266 cells were treated with 100 μM of DHAP for the time periods stated; whole-cell extracts were then prepared and examined via Western blot analysis for caspase-8 and caspase-9; (**F**) U266 cells were treated with 100 μM of DHAP for the time periods stated; whole-cell extracts were then prepared and analyzed via Western blot analysis for caspase-3 and PARP. To confirm equal protein loading, the immunoblot was stripped and reprobed for β-actin. Densitometric quantitation in fold change of each band has been indicated below the gel. * *p* < 0.05, ** *p* < 0.01, *** *p* < 0.001, vs. control.

To determine if DHAP affects cell proliferation in U266 cells, we used flow cytometry to evaluate its effect on cell cycle distribution. As shown in Figure 2A, DHAP triggered a powerful G2/M phase arrest in a time-dependent fashion, concomitant with growth inhibitory effects (Figure 2D) in the U266 cells. We next evaluated the apoptosis-triggering effects of DHAP in U266 cells, and discovered that DHAP caused increases in the number of apoptotic cells , as determined by the Annexin V (Figure 2B) and TUNEL staining assays (Figure 2C). To define the mechanism of DHAP-induced apoptosis in U266 cells, we used Western blot analysis to examine the effect of DHAP (100 μM) treatment of U266 cells. As shown in Figure 2E,F, time-dependent apoptosis induced by DHAP was confirmed by cleavage of caspase-3, caspase-8, caspase-9, and poly (ADP-ribose) polymerase (PARP).

### 2.3. DHAP Activates MAPK Signaling Pathways

**Figure 3:**
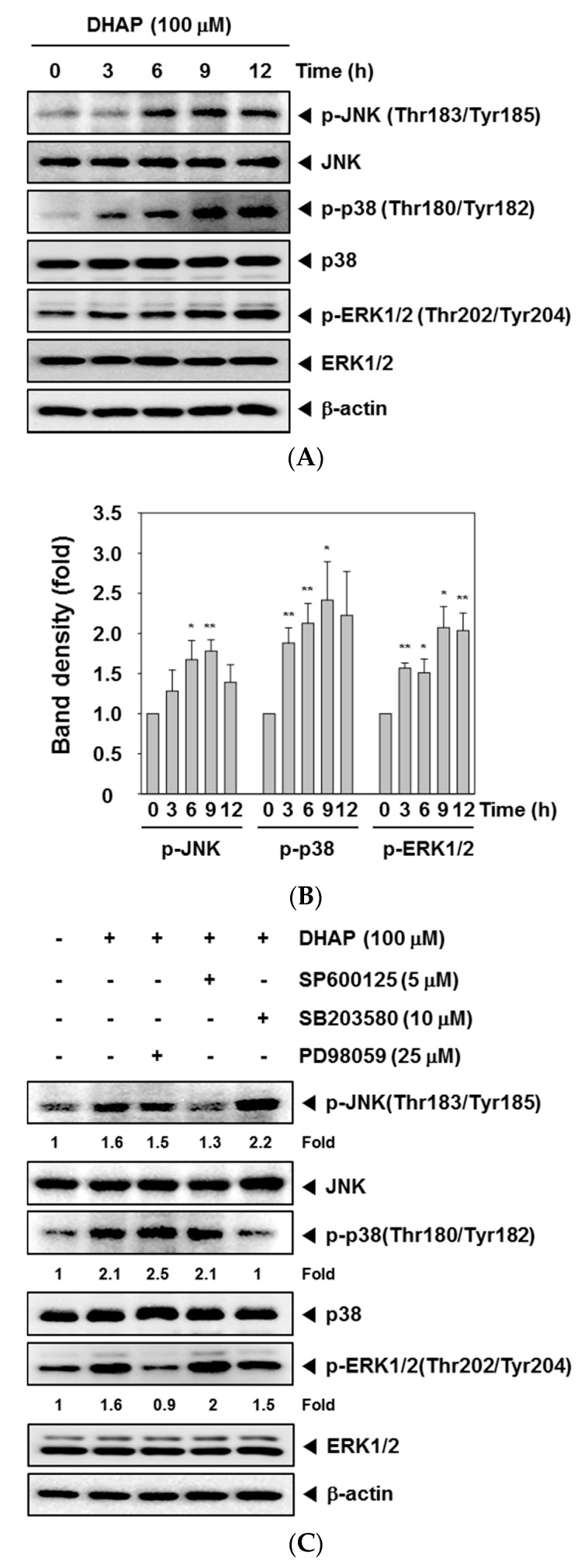

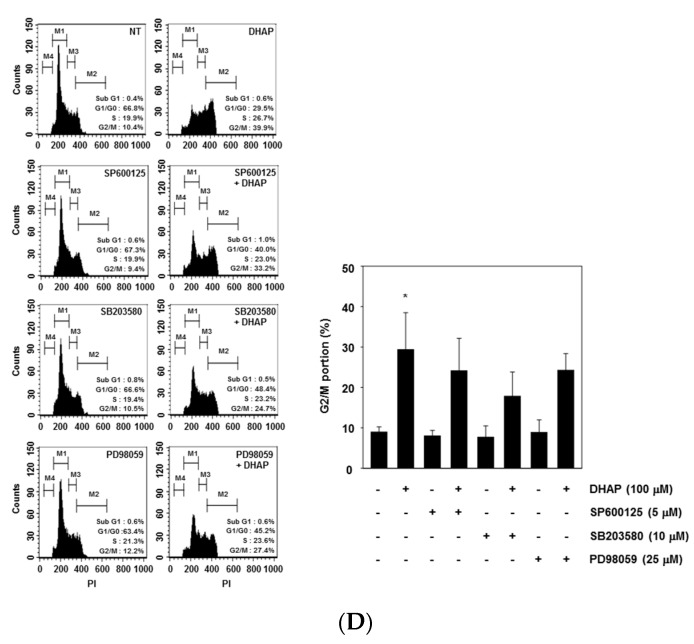
Effect of DHAP on MAPK activation in U266 cells. (**A**) The cells were treated with 100 μM of DHAP for the time periods stated; whole-cell extracts were then prepared and analyzed via Western blot analysis for p-p38 (Thr180/Tyr182), p-JNK (Thr183/Tyr185), and p-ERK1/2 (Thr202/Tyr204) by Western blot analysis. To confirm equal protein loading, the immunoblot was stripped and reprobed for JNK, p38, and ERK1/2. The results shown are representative of the three independent experiments; (**B**) The ratios of phosphorylated proteins to non-phosphorylated proteins were measured and the band density values were expressed as mean ± SE; (**C**) U266 cells were pretreated with SP600125 (5 μM), SB203580 (10 μM), or PD98059 (25 μM) for 30 min, then incubated with DHAP (100 μM) for 12 h. The expression of p-p38 (Thr180/Tyr182), p-JNK (Thr183/Tyr185), and p-ERK1/2 (Thr202/Tyr204) was ascertained via Western blot analysis. To confirm equal protein loading, the immunoblot was stripped and reprobed for total JNK, p38, and ERK1/2 proteins. Densitometric quantitation in fold change of each band has been indicated below the gel; (**D**) U266 cells were pretreated with SP600125 (5 μM), SB203580 (10 μM), or PD98059 (25 μM) for 30 min, then incubated with DHAP (100 μM) for 48 h. Cellular DNA staining with PI and flow cytometric analysis was performed to ascertain the cell cycle distribution. * *p* < 0.05, ** *p* < 0.01, vs. control.

MAPK signaling pathways have a significant role in cancer tumorigenesis [24]. We therefore conducted Western blot analysis to check if DHAP could modulate the activation of MAPK, including p38, JNK, and ERK in tumor cells. As displayed in Figure 3A,B, DHAP substantially induced the phosphorylation of p38, JNK, and ERK within U266 cells. When the cells were pretreated with p38 inhibitor SB203580 (10 μM), JNK inhibitor SP600125 (5 μM), or ERK inhibitor PD98059 (25 μM) for 30 min, DHAP-induced p38, JNK, and ERK activation were found to be blocked in pharmacological inhibitor-treated cells, respectively (Figure 3C). We also found that SP600125, SB203580, and PD98059 attenuated DHAP-induced G2/M phase arrest in U266 cells (Figure 3D).

### 2.4. DHAP Causes Potentiation of the Apoptotic Effect of Bortezomib in U266 Cells

**Figure 4:**
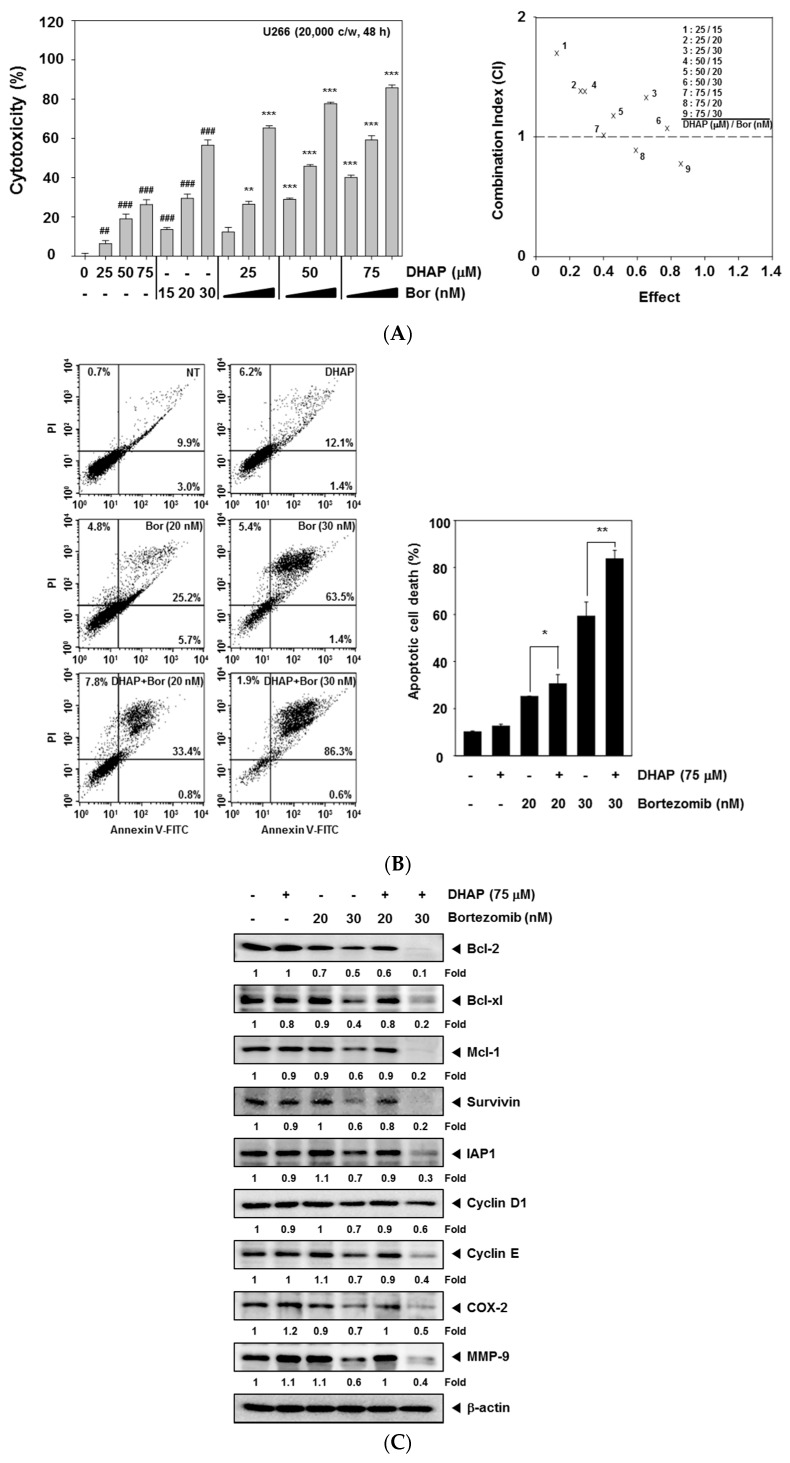

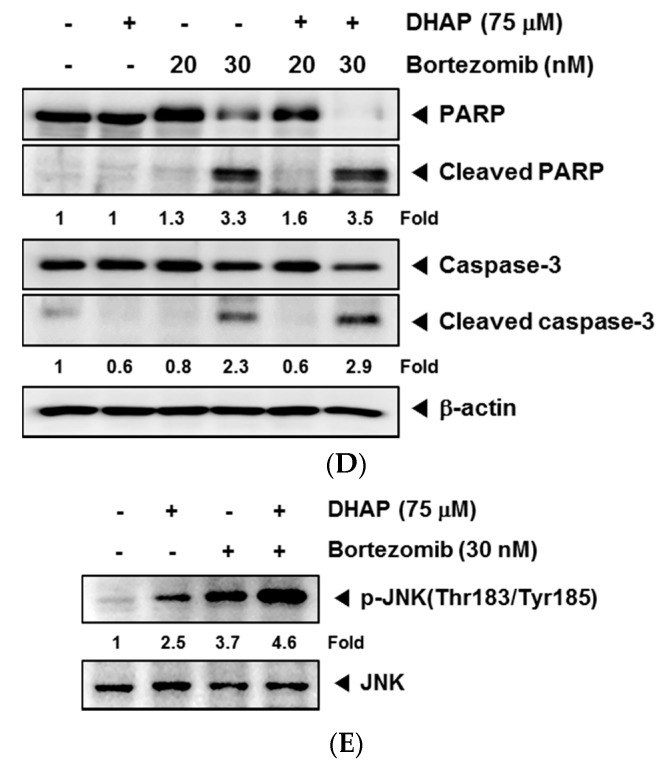
Effect of DHAP on bortezomib-induced apoptosis in U266 cells. (**A**) The cells were treated with DHAP (0, 25, 50, and 75 μM) and bortezomib (0, 15, 20, and 30 nM) for 48 h. MTT assays were used to determine the cytotoxicity (*left*). The DHAP synergistically enhanced the bortezomib-induced cell death in U266 cells (*right*). The average of the CI values was calculated from nine separate combinations. A CI of less than 1 was regarded as synergistic, a CI of 1 was regarded as additive, and a CI greater than 1 was regarded as antagonistic. U266 cells were treated simultaneously with 75 μM of DHAP and 20 and 30 nM of bortezomib for 48 h; (**B**) The cells were incubated with an FITC-conjugated Annexin V, then analyzed with flow cytometry for apoptotic effect; (**C**) Bcl-xl, Bcl-2, Mcl-1, Survivin, IAP1, COX-2, Cyclin D1, Cyclin E, and MMP-9 protein levels were determined by Western blot analysis; (**D**) PARP and caspase-3 protein levels were ascertained via Western blot analysis. To confirm equal protein loading, the immunoblot was stripped and reprobed for β-actin; (**E**) U266 cells were treated simultaneously with 75 μM of DHAP and 30 nM bortezomib for 12 h, following which whole-cell extracts were created and examined for p-JNK (Thr183/Tyr185) via Western blot analysis. To confirm equal protein loading, the immunoblot was stripped and reprobed for total JNK protein. Densitometric quantitation in fold change of each band has been indicated below the gel. ^##^ *p* < 0.01, ^###^ *p* < 0.001, vs. control, * *p* < 0.05, ** *p* < 0.01, *** *p* < 0.001, vs. bortezomib alone.

We next determined if DHAP could enhance anti-MM agent bortezomib-induced cell death. The cytotoxicity of the combined treatment was analyzed via MTT assay, and the presence of synergistic effects was determined using CalcuSyn software (Biosoft, Cambridge, UK). As shown in Figure 4A, the combination index (CI) indicated that certain combinations of DHAP and bortezomib (75 μM DHAP/20 nM and 30 nM bortezomib) synergistically inhibited U266 cell growth. We then tried to determine if DHAP could cause potentiation of the apoptotic effects of bortezomib within U266 cells via Annexin V assay, and found that DHAP substantially enhanced the apoptotic effect of bortezomib in U266 cells (Figure 4B). We found that treatment of cells with the combination of DHAP and 30 nM bortezomib resulted in a marked attenuation of the levels of expression of Bcl-xl, Bcl-2, Mcl-1, Survivin, IAP1, Cyclin D1, Cyclin E, MMP-9, and COX-2 in U266 cells (Figure 4C). Additionally, the levels of PARP and caspase-3 cleavage were elevated upon the co-treatment of DHAP and bortezomib within U266 cells (Figure 4D). We also found that a treatment employing a combination of DHAP and bortezomib significantly increased JNK activation, compared with treatment with individual agents alone (Figure 4E). These results show that combination treatment of cells results in increased apoptosis.

## 3. Discussion

The objective of this research was to determine the anti-cancer properties of DHAP in multiple myeloma cells. It was observed that DHAP downregulated the expression of certain gene products, including Bcl-xl, Bcl-2, Mcl-1, Survivin, IAP1, Cyclin D1, Cyclin E, MMP-9, and COX-2, with concomitant up-regulation of Bax and p21 expression. DHAP caused G2/M phase arrest, inhibition of proliferation, induction of apoptosis, and led to activation of caspase-3, caspase-8, and caspase-9. We also found that DHAP induced the activation of JNK, p38, and ERK, and caused substantial potentiation of bortezomib’s apoptotic effects in U266 cells.

It has recently been demonstrated that the apoptosis signaling systems constitute potentially good targets for the development of new anticancer agents [25]. Understanding how DHAP exerts its anticancer effects is therefore important for developing it for cancer prevention and/or treatment. Our results show that the anti-cancer effect of DHAP involves induction of apoptosis. DHAP-induced apoptosis in multiple myeloma cells was detected by Annexin V staining and TUNEL staining (DNA fragmentation), and further confirmed by the activation of caspase-3, caspase-8, and caspase-9, and PARP cleavage. It was discovered that DHAP inhibited the expression of several gene products linked to the initiation and promotion of tumors. These products include anti-apoptotic (Bcl-xl, Bcl-2, Mcl-1, IAP1, and Survivin), proliferation (Cyclin D1 and Cyclin E), and metastatic (COX-2 and MMP-9) gene products. The down-regulation of Bcl-xl, Bcl-2, IAP-1, Survivin, and Mcl-1, and the upregulation of the expression of pro-apoptotic protein Bax, could play a part in DHAP’s ability to trigger apoptosis within U266 cells. The down-modulation of the expression of MMP-9 and COX-2 may account for the anti-metastatic effects of DHAP, which requires subsequent research.

The inhibition of cell proliferation and/or the induction of apoptosis are strongly linked to the activation of several intracellular signaling pathways resulting in cell cycle arrest in the S, G1, or G2/M phase [26,27,28]. The downregulation of the expression of cyclin D1 and cyclin E by DHAP is associated with the suppression of proliferation and cell cycle arrest at the G2/M phase, suggesting that cyclin D1 and cyclin E may play a part in DHAP-induced G2/M phase arrest, resulting in the restriction of cell growth and possible apoptotic cell death. It has been suggested that Cyclin E is an attractive target for molecular therapeutics, both because it is overexpressed in a significant fraction of human tumors [29] and this overexpression has been implicated in tumor progression [30]. It has been postulated that interference with cyclin E expression could inhibit the neoplastic growth of a variety of cancers [29]. The increased expression of p21 is linked to inhibition of the cell cycle, apoptosis, and differentiation [31], and the induction of p21 subsequently results in the arrest of the cell cycle’s G1/G0 or G2/M phase [32,33]. We found that DHAP increased the p21 expression in U266 cells, and this up-regulation of p21 could be one molecular mechanism via which DHAP restricted the growth of tumor cells and triggered cell cycle arrest.

It has been demonstrated by recent studies that MAPK signaling pathways modulate cell cycle arrest. The activation of ERK1/2, JNK, and p38 MAPK signaling has been shown to be essential for G2/M phase arrest [34,35,36]. Our results show that pretreatment with p38 inhibitor SB203580, JNK inhibitor SP600125, and ERK1/2 inhibitor PD98059 attenuated DHAP-induced G2/M phase arrest. Therefore, it is likely that DHAP induced G2/M phase arrest in MM cells by activating the MAPK pathway. In addition, it is possible for MAPK pathways to be activated differentially, and their exact connection to apoptosis depends significantly on cell type as well as the stimuli used. The p38 pathways and JNKs are generally associated with an increased level of apoptosis, while the ERK1/2 pathway has been shown to block apoptosis [37,38,39]. However, several studies have reported the involvement of ERK1/2 in certain varieties of chemotherapeutic or preventive agent-induced apoptosis, such as quercetin, resveratrol, and taxol [40,41,42]. We found that DHAP also induced the substantial activation of all three MAPKs in U266 cells. These results suggest that DHAP may act on the activation of the MAPK signaling pathway to induce apoptosis and cell cycle arrest, and inhibit the proliferation of MM cells.

Bortezomib is a proteasome inhibitor in clinical use for treating multiple myeloma [22,43,44,45]. For this study, DHAP was observed to inhibit cell growth and induce apoptosis of MM cells by activating MAPK signaling pathways. Therefore, DHAP was used in combination with bortezomib to explore its potential to enhance therapeutic efficacy. It was observed that DHAP did indeed significantly enhance bortezomib’s apoptotic effects in U266 cells, as indicated by MTT and Annexin V assays, and the downregulation of various gene products that mediate tumor cell survival, proliferation, and metastasis. Furthermore, combination treatment resulted in greater phosphorylation of JNK, compared with DHAP or bortezomib alone. Overall, our results demonstrate that DHAP inhibited the proliferation of tumor cells by causing apoptosis and G2/M phase arrest. DHAP-induced cell cycle arrest and apoptosis were linked to the activation of the MAPK pathway.

## 4. Materials and Methods

### 4.1. Reagents

2,5-Dihydroxyacetophenone (DHAP), 3-(4,5-dimethylthiazol-2-yl)-2,5-diphenyltetrazolium bromide (MTT), propidium iodide (PI), and RNase A were obtained from Sigma-Aldrich (St. Louis, MO, USA). DHAP was dissolved as a 50 mM stock solution in dimethyl sulfoxide, and kept at −20 °C. Additional dilution was performed in a cell culture medium. Fetal bovine serum (FBS), RPMI 1640, and an antibiotic–antimycotic mixture were obtained from Thermo Fisher Scientific Inc. (Waltham, MA, USA). Antibodies against Bcl-2, Bcl-xl, β-actin, Mcl-1, IAP-1, Survivin, COX-2, MMP-9, Bax, p21, PARP, Caspase-3, and HRP-conjugated secondary antibodies were purchased from Santa Cruz Biotechnology (Santa Cruz, CA, USA). Antibodies against JNK, p-JNK (Thr183/Tyr185), p38, p-p38 (Thr180/Tyr182), p-ERK1/2 (Thr202/Tyr204), ERK1/2, Cyclin D1, Cyclin E, Caspase-8, and Caspase-9 were obtained from Cell Signaling Technology (Beverly, MA, USA).

### 4.2. Cell Lines and Cell Culture

Human multiple myeloma cell line U266 cells were purchased from the American Type Culture Collection (Manassas, VA, USA). U266 cells were cultured in RPMI 1640 incorporating 10% FBS, 100 μg/mL of streptomycin, and 100 units/mL of penicillin. The cells were kept at 37 °C in an atmosphere of 5% CO_2_.

### 4.3. Western Blot Analysis

U266 cells were treated with various concentrations of DHAP for various periods, then lysed; their protein concentrations were revealed with a Bradford reagent (Bio-Rad, Hercules, CA, USA). Whole-cell extracts were isolated at 10–12% sodium dodecyl sulfate-polyacrylamide gel electrophoresis (SDS-PAGE), then electro-transferred to nitrocellulose membranes (Pall Corporation, MI, USA); these membranes were subsequently blocked with 5% nonfat milk or 2% BSA in Tris-buffered saline with 0.1% Tween 20 (TBST) for 2 h at room temperature, then incubated overnight with the respective primary antibodies at 4 °C. Following this, the membranes were washed and incubated with HRP-conjugated secondary antibodies (1:5000) at room temperature for 2 h; the membranes were then detected using an enhanced chemiluminescence (ECL) kit (Millipore, Bedford, MA, USA). The statistical analysis and densitometry values for the Western blot analysis were performed by the Sigma plot (Systat Software, Inc., San Jose, CA, USA) and Image J software (National Institutes of Health, Bethesda, MD, USA).

### 4.4. Reverse Transcription Polymerase Chain Reaction (RT-PCR)

Total RNA was extracted with a Trizol reagent, in accordance with the instructions from the manufacturer (Invitrogen, Carlsbad, CA, USA). One microgram of total RNA was changed to cDNA via reverse transcriptase, then amplified with a Taq polymerase via the use of an RT-PCR kit (Takara Bio Inc., Tokyo, Japan). The relative expressions of *Bcl-xl*, *Bcl-2*, *Cyclin D1*, *Survivin*, *MMP-9*, *COX-2*, *Bax*, and *p21* were analyzed using a TaKaRa PCR Thermal Cycler (Code TP350, Takara Bio Inc., Tokyo, Japan) with glyceraldehyde-3-phosphate dehydrogenase (*GAPDH*) as an internal control. The reaction was initially run for 5 min at 94 °C, followed by 30 cycles of 30 s at 94 °C, 30 s at 55–60 °C, and 1 min at 72 °C, with a final period of 10 min at 72 °C. PCR products were run on 1% agarose gel, after which they were stained with loading star (Dynebio, Gyeonggi, Korea). The stained bands were visualized under UV light and then photographed.

### 4.5. MTT Assay

The anti-proliferative effect of DHAP was ascertained by MTT assay. U266 cells were seeded in 96-well plates at a density of 1 × 10^4^ cells/well, and incubated with 50 and 100 μM of DHAP. After 12, 24, 36, and 48 h incubation, 30 μL of MTT solution (2 mg/mL) was added to each well. After incubation at 37 °C for 2 h, 100 μL of extraction buffer (20% SDS and 50% dimethylformamide) was added to the cells. The cells were then incubated at 37 °C overnight, following which the absorbance was measured at 570 nm via a microplate reader (Bio-Rad, Hercules, CA, USA).

### 4.6. Cell Cycle Analysis

The effect of DHAP on the distribution of cells in different phases of the cell cycle was analyzed by flow cytometry. U266 cells were seeded on 6-well plates, at a density of 1 × 10^6^ cells/well. The cells were treated for 24 and 48 h with 100 µM of DHAP, then collected and washed with PBS. Cell pellets were fixed overnight at −20 °C in 70% cold ethanol. The fixed cells were then re-suspended in PBS incorporating 1 mg/mL of RNase A, and incubated at 37 °C for 1 h. Following this, the cells were washed, re-suspended, and stained in PBS containing 25 µg/mL of PI at room temperature in darkness for 30 min. Finally, the DNA contents of the stained cells were examined using Cell Quest 3.0 Software with FACScan Calibur flow cytometry (BD Biosciences, Becton-Dickinson, Franklin Lakes, NJ, USA). Each cell cycle phase is indicated as M1, M2, M3, and M4. M1 corresponds to G1/G0 phase, M2 for G2/M phase, M3 for S phase, and M4 for sub G1 phase.

### 4.7. Annexin V Assay

Early apoptotic cell death was determined with an Annexin V-FITC Apoptosis Detection Kit (Bio-Rad, Hercules, CA, USA), in accordance with the instructions from the manufacturer. One early sign of apoptosis is the swift translocation and accumulation of the membrane phospholipid phosphatidylserine from the cytoplasmic interface of the cell to the extracellular surface. This loss of membrane asymmetry can be ascertained with the binding properties of Annexin V. U266 cells were plated in 6-well plates at a density of 1 × 10^6^ cells/well, and were then treated with 100 μM of DHAP for 24 and 48 h. Following the treatment, the cells were stained with annexin V conjugated to fluorescein isothiocyanate (FITC), or with PI. The stained samples were then analyzed using Cell Quest 3.0 software with a flow cytometer.

### 4.8. TUNEL Assay

Late apoptotic cell death was ascertained with a TUNEL (terminal transferase mediated dUTP-fluorescein nick end labeling) assay kit (Roche Diagnostics GmbH, Penzberg, Germany), in accordance with the instructions from the manufacturer. In brief, U266 cells were treated with 100 µM of DHAP for 24 and 48 h, then washed with cold PBS. The cells were seeded after being fixed with 4% paraformaldehyde for 30 min and washed twice with PBS. Following this, the resuspended cells were placed in a permeabilization solution (0.1% Sodium citrate and 0.1% Triton X-100) for 20 min at 4 °C, then washed with cold PBS. The cells were subsequently incubated with a TUNEL label mixture and TUNEL enzyme for 1 h at 37 °C in darkness. Finally, the cells were washed with PBS and analyzed using Cell Quest 3.0 software with a flow cytometer.

### 4.9. Statistical Analysis

All the numerical values are displayed as the mean ± SD. The statistical significance of the data compared with the untreated control was ascertained using the Student’s *t*-test. Significance was fixed at *p* < 0.05.
